# Supplementary material for: Quantitative evaluation of choriocapillaris using optical coherence tomography and optical coherence tomography angiography in patients with central serous chorioretinopathy after half-dose photodynamic therapy
Source: PLoS One. 2020 Jan 13;15(1):e0227718. doi: 10.1371/journal.pone.0227718 (PMC6957295; doi:10.1371/journal.pone.0227718)
Supplement: Attachment — Submitted filename: 1._response_to_reviewers.docx [file pone.0227718.s002.docx]

**Reviewer #1**:

1. The authors evaluated choriocapillaris changes by evaluating flow voids and choroidal thickness in chronic CSC treated with half dose PDT and evaluated the patients at 1, 3 and 6 months after treatment.
It is not clear why the mean follow- up was 4.4months+/-1.8 months if all the patients were evaluated after 6 months. The classification of chronic CSC is not quite clear. The authors included patients that after three months of CSC did not spontaneous resolution? Is it correct? Usually 6 months are considered a chronic CSC.

We would like to thank Reviewer #1 for their evaluation and the feedback. Regarding the follow-up, it was the pretreatment follow up where we expected at least 3 months of symptom duration. The exact duration of symptoms is not easily assessable at all times. We have amended the manuscript for more clarification. The pretreatment follow up was: symptom duration > 3 months + observation duration > 3 months (4.4 m).

We have therefore added “symptom duration was at least 6 months before treatment.” in the abstract, provided further clarification of “chronic CSC (at least 6 months including first symptom)” in the “Patients” section in material and methods, and added the following line “The patients had had visual symptom of decreased vision, metamorphopsia, and micropsia and average pretreatment symptom duration was 9.2 ± 1.6 months” in the results section.

Finally, we only included patient who received half-dose PDT for foveal subretinal retinal detachment as described in “Patients’” section in material and methods.

2. There is no control group in this study. This is a significant limitation of this retrospective study.

We believe that the control group in an interventional longitudinal study is not essential. In addition, we conducted a multivariate analysis in order to remove the confounding factors. However, the control group such as the self-resolution group within 6 months without treatment is much better to show the findings in our study. For further clarification regarding the control group exclusion, we have addressed this in the limitation section.

3. In the introduction, please update the available treatments for CSC with subthreshold micropulse laser.

We have updated the “subthreshold micropulse laser” in the Introduction and its references.

4. In the methods section, the choriocapillaris flow voids evaluation should be better explained. Did the authors use the method proposed by Zhang et al (IOVS 2018)? It should be better explained how the artifacts due to the presence of fluid were corrected?

We have looked up the papers from Spaide (AJO 2016), Rochepeau at al (AJO 2018), and Zhang et al (Ophthalmol Retina 2017 and IOVS 2018), and added an explanation of the flow signal void in the method section. Please see below or the manuscript for he updated section for more details;

“In the previous study [32], the difference in compensation rate was not substational showing less than 10% of total amount of correction when using flow signal voids with the Phansalkar method of reducing the shadowing artifact from superimposed retinal structure. Spectralis provides an EDI mode of OCTA while HEYEX provides the projection artifact removal mode for a better analysis of the outer retina and choroidal structures. Even when using these methods, the shadowing effect of the subretinal fluid and RPE attenuation cannot be fully controlled by image modulations. One of the best way to control the compounding factors (retinal thickness for subretinal fluid and RPE thinning, BCVA-related to the integrity of the photoreceptor and RPE layer, choroidal thickness, age etc.) is the statistical correction using multivariate analysis with related variables (flow signal voids, retinal thickness, age, sex, BCVA, subfoveal choriocapillaris layer thickness, etc.). In addition, we supplied all data with or without SRF and conducted subgroup analysis for checking the effect of SRF. Furthermore, the increased CST was found to be related in the decreased flow signal void in all data whereas decreased CST was related in the increased flow signal void. The flow signal void decreased while the CST thinned without the effect of photoreceptor and RPE thinning. This indicates that the flow signal is mainly affected by not the image artifact but thechronic CSC itself. However, further validation of the statistical correction of compounding factors is required.”

**Reviewer #2:**

Seung Yang et al. present a retrospective analysis of changes in the choriocapillaris and choroid following PDT in patients with CSC by means of OCT and OCT-A. Though the idea behind this study is very interesting and relevant, there are several issues needing clarification. Please find a more detailed description in the comments below.
1. Abstract: Results: the number of patients/ eyes is missing and should be mentioned here.
It is not clear, for what time interval the results and p-values are presented – for 1, 3 or 6 months?

We would like to thank Reviewer #2 for their evaluation and the feedback. Regarding their comment, have we added the number of patients and in the abstract. We have also clarified which intervals the p-values have been presented for in the abstract.

2. Definition of chronic CSC: The authors define “chronic” CSC as CSC with symptoms >3months. The common definition of “chronic”, however, is a duration of symptoms/ disease >6 months.

For more clarification regarding the “chronic” condition we have changed the explanation.

Regarding the follow up, it was the pretreatment follow up where we expected at least 3 months of symptom duration. The exact duration of symptom is not sometimes difficult to know. We have amended the manuscript for more clarification. The pretreatment follow up was: symptom duration > 3 months + observation duration> 3 months (4.4 m).

We have therefore added “symptom duration was at least 6 months before treatment.” in the abstract, provided further clarification of “chronic CSC (at least 6 months Including first symptom)” in the “Patients” section in material and methods, and added the following line “The patients had had visual symptom of decreased vision, metamorphopsia, and micropsia and average pretreatment symptom duration was 9.2 ± 1.6 months” in the results section.

Finally, we only included patient who received half-dose PDT for foveal subretinal retinal detachment as described in “Patients’” section in material and methods.

3. The illustration of in- and exclusion criteria is confusing, a better structure is needed here.

We have updated the manuscript with the following explanation in the methods;

“This retrospective case series included 91 consecutive treatment naïve patients (age, >20 years) with chronic CSC (lasting longer than 6 months and within the 12 months after first symptom onset) without severe atrophy who received half-dose PDT for foveal subretinal serous detachment between January 1, 2016 and July 31, 2018. The study was conducted at the Ophthalmology Department of Veterans Health Service Medical Center and Seoul Shinsegae Eye Center. The diagnosis was based on the patient’s symptom, visual acuity, fundoscopic examination, fluorescein angiography, indocyanine green angiography, Enhanced depth imaging (EDI)-OCT (Spectralis, Heidelberg Engineering, Heidelberg, Germany), and OCTA (Spectralis, Heidelberg Engineering, Heidelberg, Germany). Subjects were excluded if they had a symptom duration of fewer than 6 months, any sign of choroidal neovascularization in fluorescein angiography, indocyanine green angiography and OCTA, previous history of PDT, focal laser photocoagulation, or anti-VEGF treatment, evidence of choroidal atrophy or neovascularization, high myopia defined as a refractive error (spherical equivalent) < −6.0 diopters or an axial length > 26.5 mm, underwent continuous corticosteroid therapy, have a history of intraocular surgeries, poor signal strength, or severe artifacts due to saccadic eye movement. ”

4. Were OCT-A - CC images averaged? This may be crucial for further (quantitative) analysis.

Yes the 5 images were averaged in ART mode. We have added an explanation regarding this in the methods section.

5. Details on OCT-A image acquisition are missing: were these 3mm x 3mm images (the authors mention a 9mm² scan)? How many scans were used? Were the scans centered on the fovea? And if so, in a quite small test field of 9mm² , in some cases the “area of interest” was probably not (completely) included within these scans – how did the authors ensure that this was the case?
Why did the authors solely analyze the choriocapillaris and not the choroid (e.g. as inner, mid and outer choroid sections)?

The size was 4.5mm x 3 mm. The number of B-scan was 256 and the distance between B-scans was 11 um. The scans centered on the fovea by detecting fixation with AutoRescan. The AutoRescan feature of the Spectralis OCT device provides an OCT scan and a corresponding high-quality fundus picture and relies on active eye tracking.

We have updated the manuscript with relevant information in the method’s section.

6. Statistics: t-test: How do the authors presume their data to be normally distributed?

It is said that Central Limit Theorem “kicks in” at an N of about 30. In other words, as long as the sample is based on 30 or more observations, the sampling distribution of the mean can be safely assumed to be normal. Furthermore, for larger datasets (say N>40), the Central Limit Theorem suggests that the t-test will produce valid results even in the face of non-normally distributed data In our case, the N was 56 and the shape of distribution appeared to follow the normal distribution. However, for more accuracy, the histogram checking and the Kolmogorov-Smirnov test might be used. Therefore, we checked every data in baseline characteristics at baseline and 6 months, and the quantitative values of OCT and OCTA variables at baseline, 1 month, 3 months, and 6 months. The histogram checking and Kolmogorov–Smirnov test reported that the all data associated with the variables in this study was approximately normally distributed

With this in mind, we added the following sentences in the methods section.

“All data are presented as the mean ±standard deviation. When comparing 2 groups, either the independent t-test or the chi-square test was used depending on the number of samples and whether the assumption of a normal distribution was met. The normal distributions of baseline characteristics of patients and the parameters in OCT and OCTA were checked using the histogram and the Kolmogorov–Smirnov test. ”

7. The measurement of IOP is mentioned in the results section, but not in the methods section.

We have updated the methods section accordingly with information on the “intraocular pressure using Goldmann applanation tonometry”.

8. Figure 2: This is in the reviewer’s opinion the most important issue in this manuscript: How can the authors exclude that the observed phenomenon (decrease of flow voids in OCT-A) is caused by a “shadowing” artifact that is induced by superimposing structures (e.g. subretinal fluid) and thus decreases when the fluid diminishes? Have the authors thoroughly looked at all retinal layers and the ORCC-layer for possible causes of shadowing artifacts and morphological changes between pre- and posttreatment (in the OCT-A images in Fig 2-A and B, there is for instance a visible shadowing artifact from superimposing retinal vessels)? Have the authors applied corrections for projection artifacts? Could the presence of neovascular membranes be definitely excluded? Quaranta-El Maftouhi et al, (AJO, 2015) describe small hyperreflective undulations of the RPE in OCT-scans of patients with chronic CSC, that were shown to represent neovascular complexes. In the OCT-scan shown in figure 2-B, we can also see such small hyperreflective undulations of the RPE…
Further, the OCT B-scan in Figure 2-A shows shadowing artifacts at the level of the RPE/ CC induced by hyperreflective structures at the level of the elevated outer retina, while this is not visible in Figure 2-B.

Please see the response to question #4 of reviewer 1, As you can observe in the figure 2, the decreased flow signal void is not confined to the area of SRF, but the outside of SRF area.

Regarding this, we added the following sentences in the limitation section of the discussion.

“In addition, there are major issues about shadowing effect regarding SRF, photoreceptor layers disruption, and RPE thickening and thinning. In the previous study [32], the difference in compensation was not critical less than 10% of total amount of correction when using flow signal voids with the Phansalkar method of reducing the shadowing artifact from superimposed retinal structure. Spectralis provides an EDI mode of OCTA while HEYEX provides the projection artifact removal mode for a better analysis of the outer retina and choroidal structures. Even when using these methods, the shadowing effect of the subretinal fluid and RPE attenuation cannot be fully controlled by image modulations. One of the best way to control the compounding factors (retinal thickness for subretinal fluid and RPE thinning, BCVA-related to the integrity of the photoreceptor and RPE layer, choroidal thickness, age etc.) is the statistical correction using multivariate analysis with related variables (flow signal voids, retinal thickness, age, sex, BCVA, subfoveal choriocapillaris layer thickness, etc.). In addition, we supplied all data with or without SRF and conducted subgroup analysis for checking the effect of SRF.Furthermore, the increased CST was found to be related in the decreased flow signal void in all data whereas decreased CST was related in the increased flow signal void. The flow signal void decreased while the CST thinned without the effect of photoreceptor and RPE thinning. This indicates that the flow signal is mainly affected by not the image artifact but thechronic CSC itself.However, further validation of the statistical correction of compounding factors is required.”

9. Further, Fig. 2 A and B do not show the same B-scans (scan 12/25 in Figure 2-A and 13/25 in Figure 2-B).

As you have highlighted, we have amended the picture 13/25 to 12/25.

10. The OCT-A images in Figure 2-A and -B do not show typical morphological features of the CC-layer, but rather look like mid-choroid sections (see for example Wang JC, TVST, 2018). Which OCT-A layers were used here?

You may find our description “The choriocapillary layer was visualized with the user setting mode that yields a 40-μm slab between 0 and 40 μm beneath the Bruch membrane” in methods section. The pattern of superficial layer and deep layer looks slightly different depending on the slab location. There are no consensuses about the slab depth. We measured the thickness at 40 μm of the slab (almost the total thickness of choriocapillaris) images and averaged them to reduce the selection bias of specific layer of CC. See below for more details

20 μm slab from BM

40 μm slab from BM

The difference between the narrow slab and the wide slab is the existence of arteriole and venule of choriocapillaris. Thus, the wide slab, which we used, contained all the layer of choriocapillaris while the narrow slab contained the anterior choriocapillaris. Thus, the morphology of the picture can be observed differently depending of the depth of the slab.

Zouache et al. Scientific reports 2016

 

Retina 2018 Matet et al.

This is our images

11. The exact size and position of the OCT-A en face image should be indicated in the IR-image.

We have followed your recommendation and included the IR images in figure 2 .
