# Supplementary material for: Quantitative evaluation of choriocapillaris using optical coherence tomography and optical coherence tomography angiography in patients with central serous chorioretinopathy after half-dose photodynamic therapy
Source: PLoS One. 2020 Jan 13;15(1):e0227718. doi: 10.1371/journal.pone.0227718 (PMC6957295; doi:10.1371/journal.pone.0227718)
Supplement: Attachment — Submitted filename: 1._response_to_reviewers.docx [file pone.0227718.s003.docx]

Reviewer #1: (No Response)

Reviewer #2: The authors did a good job in revising the manuscript, and addressed most of the issues raised by the reviewers. However, some minor issues remain to be resolved, and in terms of style and grammar, a linguistic revision would improve the readability of the manuscript.

🡺 Thank you very much. We have checked the style and grammar for better readability.

1. Page 6: lines 94-97: choroidal neovascularizations are mentioned twice as exclusion criterion

🡺 Thank you for your valuable comment. We have removed one.

2. Abstract: 56 eyes of 56 patients were considered for the analysis, but 91 patients were included in the case series – there is a discrepancy of case numbers in the main text (methods section) and the abstract (methods section). Suggest to include the numbers in the results section of the abstract (not in the methods section) for more clarity.

🡺 Thank you for your valuable comment. As per your suggestion, we included the numbers in the results section of the abstract.

3. Answer to reviewer #2, question 8:

The authors state that “As you can observe in the figure 2, the decreased flow signal void is not confined to the area of SRF, but the outside of SRF area.”

The reviewer disagrees with this statement, as the field for OCT-A evaluation is obviously chosen too small, and does not appear to include an area without SRF.

**🡺** Thank you for your valuable comment. I agree with the comment. The OCTA cannot cover all the area without SRF. We have appropriately clarified this section of the manuscript. It now states: “the decreased flow signal voids appear to be related to the SRF (right red dotted circle, Fig 2) even though improved flow signal voids were also observed in the area surrounding the SRF” (p17, lines 329-331).
